# Self-Disclosure and Post-traumatic Growth in Korean Adults: A Multiple Mediating Model of Deliberate Rumination, Positive Social Responses, and Meaning of Life

**DOI:** 10.3389/fpsyg.2022.878531

**Published:** 2022-06-30

**Authors:** Ji-Hyun Ryu, Kyung-Hyun Suh

**Affiliations:** Department of Counseling Psychology, Sahmyook University, Seoul, South Korea

**Keywords:** trauma, growth, disclosure, rumination, social responses, meaning

## Abstract

**Background:**

To explore how self-disclosure leads to post-traumatic growth (PTG) in adults who have experienced traumatic events, this study identified the relationship between self-disclosure and post-traumatic growth in Korean adults. We examined a parallel multiple mediating model for this relationship.

**Methods:**

Participants were 318 Korean male and female adult participants aged 20 years or older who had experienced trauma. We measured deliberate rumination, positive social responses, and the meaning of life as mediating variables.

**Results:**

The results revealed that the study variables positively correlated with PTG. Self-disclosure was positively correlated with deliberate rumination, positive social responses, and meaning of life. In the multiple mediating model, deliberate rumination, positive social responses, and meaning of life mediated the relationship between self-disclosure and PTG.

**Conclusion:**

Self-disclosure, deliberate rumination, positive social responses, and meaning of life play an important role in the growth of adults who have experienced traumatic events. The findings of this study should provide valuable information for future research and for mental health professionals who want to promote the PTG of their clients.

## Introduction

Disaster is a part of life, both in the past and present, and people may experience trauma. Moreover, modern people live with different risks, such as traffic accidents, that did not always exist and could be big or small; nevertheless, everyone experiences trauma. The high possibility of trauma implies that it is not only we who might be traumatized in the case of a disaster or accident, but also our family members or close acquaintances. After analyzing the number of patients treated for post-traumatic stress disorder (PTSD) in Korea over the past 5 years, the Korea National Insurance Services (2020) announced that the number had increased by 45.4% from 2015 to 2019. However, not all people who experience trauma have PTSD, and they may even mature further after experiencing trauma (Tedeschi and Calhoun, 1995). Tedeshi and Calhoun (2004) called such maturation “post-traumatic growth” (PTG) and defined it as “positive psychological change occurred as a result of struggling with highly challenging and stressful life crises (p.1).”

To ensure that trauma works positively beyond just clinically preventing it from negatively affecting PTG, many researchers have been compelled to study the variables that affect PTG. First, studies have indicated that psychosocial intervention (Roepke, 2015) as well as social support (Volgin and Bates, 2016; Peng et al., 2021) help people grow after suffering trauma. Some studies (e.g., Zhou et al., 2017) have also identified that emotional regulation can lead to PTG. It has also been shown that high resilience (Miller et al., 2020; Zeng et al., 2021) or self-efficacy (Pooley et al., 2013; Zeng et al., 2021) could lead to PTG. Recently, Zerach (2020) found that those with high tolerance to distress are likely to grow after trauma. One study (Strasshofer et al., 2018) found that individuals’ angry reactions to experiencing trauma helps PTG.

### Self-Disclosure and Rumination After Experiencing Traumas

Depending on the ruminative response styles, either PTSD or PTG may occur after experiencing trauma (Wozniak et al., 2020). People automatically experience intrusive rumination after a traumatic event, which is a natural and normal reaction to trauma. The intrusive rumination experienced after trauma is a multidimensional concept that was theorized by Martin and Tesser (1996) and conceptualized by Treynor et al. (2003), which focuses on the harm of highly stressful and traumatic events. Intrusive rumination appears for some time after experiencing a traumatic event and then progresses to more productive rumination with deliberate effort focused on dealing with the situation over time. This deliberate rumination is associated with a lower negative affect (Nolen-Hoeksema and Davis, 2004) and is likely to lead to PTG and life satisfaction (Triplett et al., 2012). Ko and Rhee (2018) found that deliberate rumination along with emotion regulation strategies mediate between intrusive rumination and PTG. Previous studies (Triplett et al., 2012; Ko and Rhee, 2018; Kim and Bea, 2019) have revealed that deliberate rumination increases PTG. Therefore, this study focused on the way in which we could induce deliberate rumination in daily life.

This study assumes that self-disclosure is a variable that could induce deliberate rumination. Self-disclosure is the act of revealing private information to others, and previous studies (Pennebaker and O'Heeron, 1984; Pennebaker and Susman, 1988) have found health benefits in disclosing traumatic events. The therapeutic effect of self-disclosure for patients with PTSD is well-known (Yeterian et al., 2017; Ko et al., 2020). Moreover, encouraging U.S. veterans diagnosed with PTSD to talk about their trauma experiences in groups to other patients with PTSD has been extremely effective (Department of Veterans Affairs/Department of Defense, 2017). Such self-disclosure is also called adaptive disclosure by PTSD experts (Litz et al., 2015). Laurenceau et al. (1998) suggested that the speaker has cognitive awareness in the process of disclosing what they have experienced to others. Thus, we can assume that deliberate rumination might have caused such awareness. The process of disclosing one’s traumatic experience to others can lead to deliberate rumination.

### Positive Social Responses and Finding Meaning of Life After Self-Disclosure

Self-disclosure can arouse feelings of intimacy as well as cognitive awareness when listeners empathize and are supportive (Laurenceau et al., 1998). Self-disclosure with positive social responses might have healing effect. Chaudoir and Fisher (2010) studied the process of self-disclosure after observing the effects of many people’s self-disclosure in social situations, and emotional social support was one of the factors that induced further self-disclosure. When adolescents have a problem-related conversation and a positive social response induces closeness and emotional intimacy in their friendships (Rose et al., 2016). Because positive emotional experiences have also been found to be important for developing identity in social relationships (van der Gaag et al., 2017), this study assumes that positive social responses after self-disclosure would promote PTG.

Another assumption that this study adopts is that discovering the meaning of life after experiencing trauma also leads to PTG. Triplett et al.’s (2012) study suggested a model in which deliberate rumination leads to PTG, and PTG leads to discovering the meaning of life, thereby creating an experience of subjective well-being. However, it is logically more reasonable that people mature because have discovered the meaning of life after experiencing trauma, and some studies (Fischer et al., 2020; Seol et al., 2021). Seol et al. (2021) have suggested that finding the meaning of life might be a cause of PTG. In their longitudinal study, meaning of life mediated the association between the effects of post-traumatic stress symptoms and PTG among navy soldiers deployed to the Gulf of Aden, Somalia. Because introspection could be included in the healing effect of self-disclosure as described by Pennebaker (2004), this study assumes that self-disclosure might lead to PTG by discovering the meaning of life.

### Research Objectives

**Figure 1:**
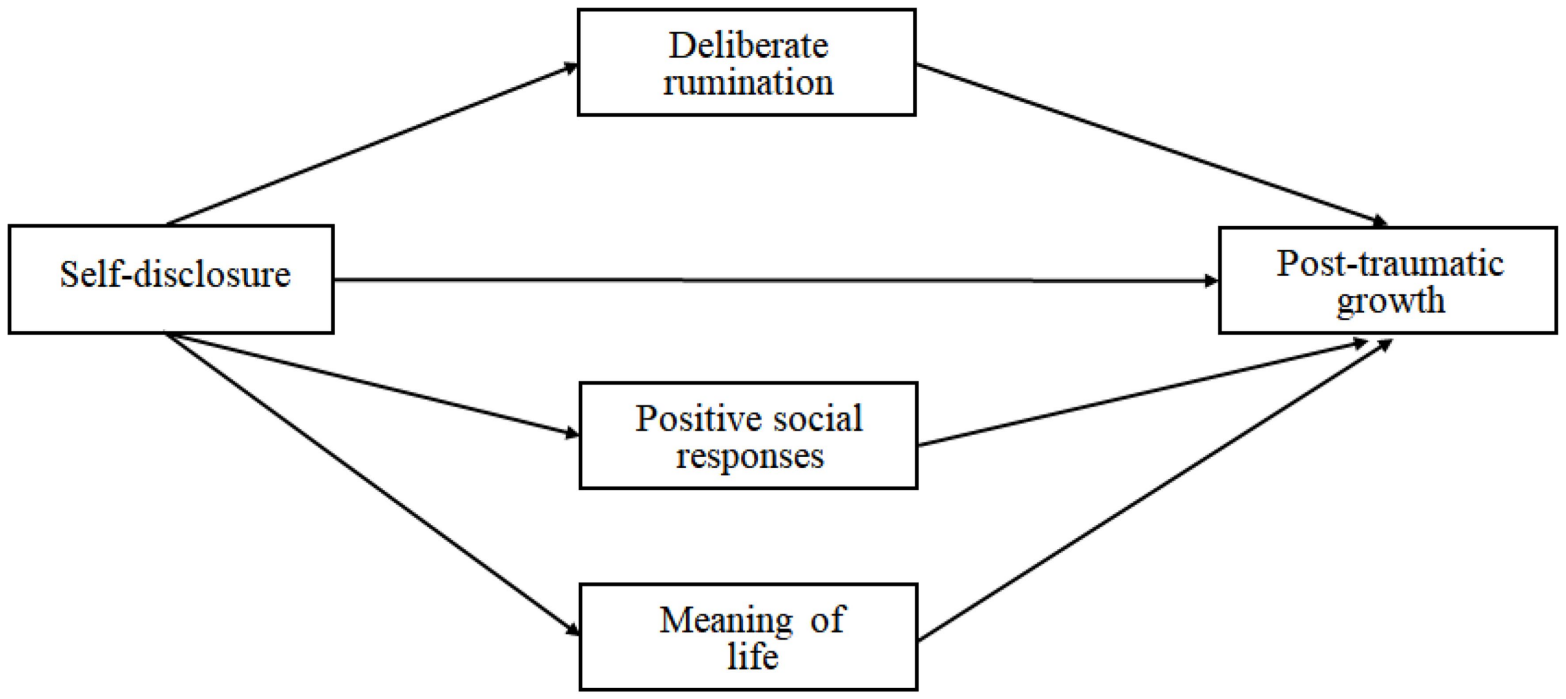
Proposed multiple mediating model.

This study aimed to identify the relationship between self-disclosure and PTG of Korean adults, and examine the mediating effects of deliberate rumination, positive social responses, and the meaning of life on the relationship. So, this study analyzed the relationships among self-disclosure, deliberate rumination, positive social responses, the meaning of life, and the PTG of Korean adults; additionally, we analyzed a multiple mediating model of deliberate rumination, positive social responses, and meaning of life on the self-disclosure and PTG of Korean adults (Figure 1). These analyses are expected to provide useful information to promote Koreans’ PTG.

## Materials and Methods

### Participants

Participants were 318 Korean male and female adults aged 20 years or older who had experienced trauma. They were selected by convenience sampling. G^*^Power 3.1.9 indicated that a minimum sample size of 307 was required to reach a statistical conclusion based on the number of predictors, a significance level of 0.001, power of 0.95, and effect size of 0.10. Thus, we planned to collect about 320 samples.

Among the participants were 210 women (66%) and 108 men (34%). The participant groups comprised 30 people (9.4%) in their 20s, 45 (14.2%) in their 30s, 164 (51.6%) in their 40s, 63 (19.8%) in their 50s, and 16 (5.0%) in their 60s or older. Regarding marital status, 63 (19.8%) were unmarried, 245 (77.0%) married, 8 (2.5%) divorced, 1 (0.3%) separated, and 1 (0.3%) answered otherwise. The most shocking traumatic events that participants reported were the death of a loved one (19.5%), followed by betrayal of a loved one (14.2%), fatal disease of a loved one (9.7%), accidents and injuries (9.1%), interpersonal breakdown (8.5%), and their own serious diseases (7.5%). Further, 236 participants (74.2%) reported experiencing negative emotions such as anxiety, fear, and depression after such traumatic events.

### Measures

#### Self-Disclosure

Participants’ self-disclosure of stress events or negative emotional experiences was measured using the self-disclosure Scale developed by Hahn et al. (2004). In the scale development study, Hahn et al. measured self-disclosure over a week, whereas we measured self-disclosure over 3 months. The frequency and depth of disclosure, and the participants’ openness in disclosing their traumatic experiences to others was rated with nine items on a seven-point Likert scale. The contents of the self-disclosure items pertained to how much the participants have disclosed about traumatic events and related emotions, how many participants have disclosed, how often the participants have confided, and how often participants have been opened about it. In the process of scale development, satisfactory reliability and validity were revealed, and the internal consistency (Cronbach’s α) of the nine items was 0.96 in this study.

#### Deliberate Rumination

Participants’ deliberate rumination related to the traumatic experience was measured with the Korean version of the Event-Related Rumination Inventory, which was developed by Cann et al. (2011) and validated by Ahn et al. (2013) for Korean people. This scale originally comprised 20 items that measured intrusive and deliberate rumination. We only assessed deliberate rumination items in this study. However, the questionnaire comprised 20 items because we measured deliberate rumination at the time of the traumatic experience and 3 weeks prior to answering the questionnaire. Items examples are, “I thought about whether I could find meaning from the event” and “I tend to think about what I felt about the experience.” Each item was rated on a seven-point Likert scale ranging from 1 (*not at all*) to 7 (*always*). The internal consistency (Cronbach’s α) was 0.94 in this study.

#### Positive Social Responses

We used the Social Reactions Questionnaire (SRQ) that was developed by Ullman (2000) and validated for Korean (K-SRQ) by Sim and Ahn (2014) to measure participants’ experience of receiving positive responses. The K-SRQ comprises 44 items and 7 subscales that measure both the positive and negative reactions that participants have received from others after disclosing traumatic experiences; only positive reaction items were used in this study. Sub-factors of positive response are emotional support (15 items) and tangible aid (3 items); however, the total score was included in the analysis. Participants rated items from 1 (*not at all*) to 7 (*always*), and the internal consistency (Cronbach’s α) of these 18 items was 0.97 in this study.

#### Meaning of Life

To measure the meaning of life perceived by participants, the Meaning in Life Questionnaire (MLQ) developed by Steger et al. (2006) was used. The MLQ used in the study was validated by Won et al. (2005) for Korean people (K-MLQ). The K-MLQ consists of 10 items and 2 subscales, presence of meaning and search for meaning. Total score was included in the analysis for multiple mediating model. Participants answered each item on a seven-point Likert scale ranging from 1 (*absolutely true*) to 7 (*absolutely untrue*). Cronbach’s α for the presence of meaning, search for meaning, and the total MLQ were 0.92, 0.94, and 0.95, respectively.

#### PTG

To measure positive changes after experiencing traumatic events among Korean adults, this study used Tedeschi and Calhoun’s (1996) PTG Inventory as validated by Song et al. (2009) for Koreans. Tedeschi and Calhoun’s original scale comprises 21 items; however, this study used the 16 items arranged by Song et al. Examples of these items are, “I found myself stronger than I thought” and “I was convinced that I could overcome the difficulties.” This scale has five subscales; however, in this study, only the total score was used in the analysis. Each item was rated using a scale ranging from 1 (*not at all*) to 7 (*always*). The internal consistency (Cronbach’s α) of the 16 items was 0.95 in this study.

#### Experiences of Traumatic Events

This study measured how severe the traumatic events were to the participants, identified their relationships with the study variables, and then determined whether they should be controlled as covariates. For this purpose, Song et al.’s (2009) Trauma Experience Questionnaire revised by Shin and Chung (2012), was used. This questionnaire comprises seven items enquiring about the type, duration, and severity of the traumatic event. Respondents were asked to report whether they experienced psychological pain and scored the severity of the subjective pain that they had experienced at the time of the traumatic event. The severity of the event was rated from 1 (*no pain*) to 7 (*very painful*).

### Procedure

This study was approved by an Institutional Review Board (IRB) before study commencement (approval number: 2-1,040,781-AB-N-01-2017117HR). Data were collected through an online survey with a questionnaire and informed consent was posted on an internet portal site, Google. This survey was promoted on social networking services, such as NAVER BAND and KakaoStory. We gave the participants disclosed research information to facilitate their understanding of this study and to encourage their participation before conducting survey. Participants were informed that they could withdraw at any time while responding to the survey. Each participants spent 20 min, on average, completing the questionnaire.

### Statistical Analysis

IBM SPSS Statistics for Windows 26.0 and PROCESS Macro 3.5 were used for all the statistical analyses. They checked not only the mean and standard deviation, but also the skewness and kurtosis of the data for parametric statistical analyses. None of the skewness absolute values exceeded 1.5, and none of the kurtosis absolute values exceeded 3.5, indicating that the variances of each variable are close to a normal distribution (Orcan, 2020).

Pearson-Product Moment correlational analysis was performed with SPSS, and parallel multiple mediating effect was performed with PROCESS Macro 3.5 model 4. Finally, bootstrapping with 5,000 resamples with 95% confidence intervals was used to analyze the significance of the indirect effects in the mediating model and to examine the differences in each of the indirect effects.

Statistical multicollinearity problems occur when tolerance is less than 0.2 or 0.1, and variance inflation factors (VIF) are greater than 5 or 10 (Kim, 2019). Because tolerances of predictors in this study were 0.739–0.918, and VIFs were 1.089–1.353, multicollinearity was not a problem. Additionally, the Durbin Watson statistic was 1.835, which indicates that there was no autocorrelation detected in the sample as it was close to 2. In addition, there were no exogenous confounding variables among demographic profiles and variables regarding traumatic experience, which was correlated with both predictors and criterion variable; thus we did not adjust the mediating model with covariates to be free from Berkson’s bias (Westreich, 2012).

## Results

### The Relationship Among the Variables Involved in PTG

Table 1 presents the results of the analysis of the relationships among the frequency of traumatic events, severity of traumatic events, self-disclosure, deliberate rumination, positive social responses, and PTG.

**Table 1:** Correlational matrix of the frequency of traumatic events, severity of traumatic events, self-disclosure, deliberate rumination, positive social responses, meaning of life, and PTG (*N* = 318).

Variables	1	2	3	4	5	6	6–1	6–2	7
1. Frequency of traumatic events	1								
2. Severity of traumatic events	0.21^***^	1							
3. Self-disclosure	0.16^**^	0.22^***^	1						
4. Deliberate rumination	0.10	0.36^***^	0.29^***^	1					
5. Positive social responses	0.06	0.17^***^	0.48^***^	0.23^***^	1				
6. Meaning of life	0.09	−0.03	0.17^**^	0.20^***^	0.24^***^	1			
6−1. Presence of Meaning	0.06	−0.07	0.15^**^	0.18^**^	0.23^***^	0.94^***^			
6−2. Search for Meaning	0.11	0.02	0.17^**^	0.19^**^	0.20^***^	0.93^***^	0.75^***^		
7. Post-traumatic growth	0.07	−0.01	0.29^***^	0.31^***^	0.38^***^	0.56^***^	0.53^***^	0.51^***^	1
*M*	3.97	9.41	35.97	81.59	84.48	56.96	28.01	28.95	81.45
*SD*	2.46	2.59	15.33	28.69	27.11	10.70	5.81	5.62	19.85
Skewness	1.40	−0.19	−0.05	0.13	−0.67	−0.90	−0.82	−1.09	−0.61
Kurtosis	3.21	−0.14	−1.14	−0.54	−0.14	0.68	0.25	1.02	−0.05

** *p* < 0.01;

*** *p* < 0.001.

The correlation analysis revealed that the frequency of traumatic events experienced by the study participants was positively correlated with self-disclosure, but not significantly to PTG (*r* = 0.07, *p* > 0.05). Additionally, the severity of traumatic events was significantly correlated with self-disclosure, deliberate rumination, and positive social responses, but there was no significant relationship between the severity of trauma events and meaning of life or PTG (*r* = −0.01, *p* > 0.05). These results suggest that the regression analysis with PTG as the criterion variable or outcome variable does not need to be adjusted by the frequency or severity of traumatic events.

Although there was a significant positive correlation between self-disclosure and PTG (*r* = 0.29, *p* < 0.001), self-disclosure was also positively correlated with deliberate rumination (*r* = 0.29, *p* < 0.001), positive social responses (*r* = 0.48, *p* < 0.001), and meaning of life (*r* = 0.17, *p* < 0.01). In addition, deliberate rumination (*r* = 0.31, *p* < 0.001), positive social responses (*r* = 0.38, *p* < 0.001), and meaning of life, (*r* = 0.56, *p* < 0.001) were positively correlated with PTG. Both presence of meaning and search for meaning were positively correlated with self-disclosure and PTG.

### Verification of Parallel Multiple Mediating Model for the PTG

**Figure 2:**
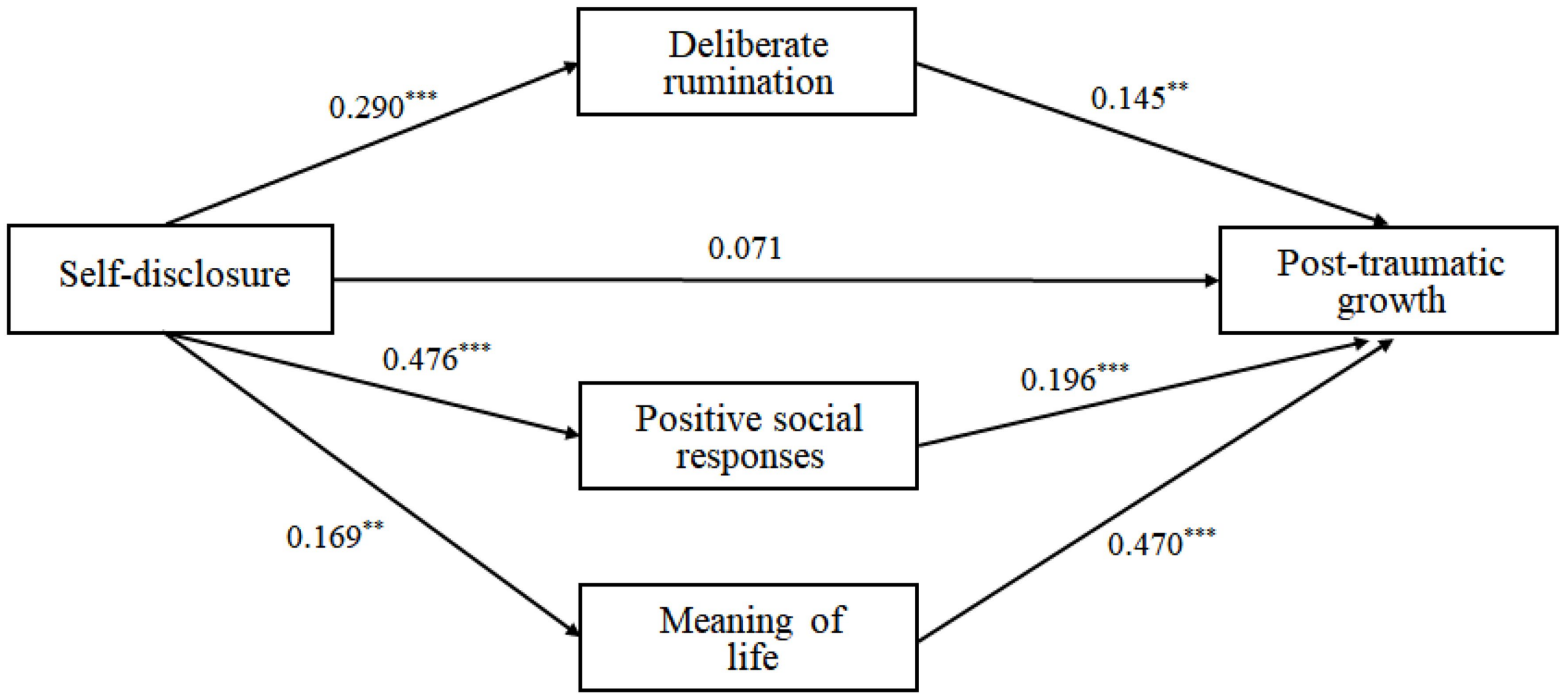
Mediating model of deliberate rumination, positive social responses, and meaning of life on self-disclosure and PTG (Standardized coefficients; ^**^p < 0.01, ^***^p < 0.001).

This study examined a parallel multiple mediating effect of deliberate rumination and positive social responses on self-disclosure and PTG (Table 2; Figure 2). The results illustrated that self-disclosure influenced deliberate rumination positively (*B* = 0.542, *p* < 0.001), and deliberate rumination had a positive influence on PTG (*B* = 0.148, *p* < 0.001). In addition, self-disclosure also affected positive social responses positively (*B* = 0.841, *p* < 0.001), and positive social responses influenced PTG positively (*B* = 0.209, *p* < 0.001).

**Table 2:** Parallel multiple mediating effects of deliberate rumination, positive social responses, and meaning of life on self-disclosure and PTG.

Variables	*B*	*S.E.*	*t*	LLCI	ULCI
**Mediating model (Outcome variable: deliberate rumination)**					
Constant	62.078	3.939	15.76^***^	54.3285	69.8279
Self-disclosure	0.543	0.101	5.38^**^	0.3442	0.7407
**Mediating model (Outcome variable: positive social response)**					
Constant	54.225	3.422	15.85^***^	47.4925	60.9584
Self-disclosure	0.841	0.088	9.61^**^	0.6690	1.0134
**Mediating model (Outcome variable: meaning of life)**					
Constant	52.722	1.514	34.83^***^	49.7430	55.7003
Self-disclosure	0.118	0.039	3.04^**^	0.0416	0.1940
**Dependent variable model (Outcome variable: PTG)**					
Constant	8.197	5.153	1.59	−1.9412	18.3351
Deliberate rumination	0.101	0.032	3.13^***^	0.0372	0.1638
Positive social responses	0.144	0.038	3.86^***^	0.0705	0.2168
Meaning of life	0.871	0.085	10.29^***^	0.7043	1.0374
Self-disclosure	0.092	0.066	1.40	−0.0376	0.2217

** *p* *< 0.01;*

*** *p < 0.001*.

*LLCI, lower level for confidence interval; ULCI, upper level for confidence interval*.

The total effect of self-disclosure on PTG is 0.370 (*p* < 0.001), the direct effect from self-disclosure to PTG was 0.092 (*p* = 0.113) and was not significant when deliberate rumination, positive social responses, and meaning of life are used as the mediating variables in this model. In Figure 2, the parallel triple mediating model indicates that self-disclosure affected deliberate rumination, deliberate rumination could promote people’s PTG, self-disclosure also affected positive social responses, and positive social responses could improve people’s PTG. In addition, self-disclosure influenced meaning of life, and meaning of life could increase PTG.

As a result of examining the indirect effects of deliberate rumination, positive social responses, and meaning of life using bootstrapping, the indirect effects were verified because there were no zeros between the upper and lower level for confidence interval of bootstrapping (Table 3). This means that self-disclosure is entirely beneficial to PTG through deliberate rumination, positive social responses, and meaning of life.

**Table 3:** Indirect effect of deliberate rumination, positive social responses, and meaning of life.

Section	Effect	*S.E.*	LLCI	ULCI
Total	0.278	0.056	0.1717	0.3943
Deliberate rumination	0.055	0.023	0.0135	0.1019
Positive social responses	0.121	0.042	0.0448	0.2125
Meaning of life	0.103	0.036	0.0364	0.1791

*LLCI, lower level for confidence interval; ULCI, upper level for confidence interval*.

Table 4 reports the differences between the indirect effect sizes that were examined using bootstrapping. Because there were zeros between the upper and lower confidence interval level of bootstrapping in deliberate rumination and positive social responses, in deliberate rumination and meaning of life, and in positive social responses and meaning of life, there were no significant differences in the indirect effect sizes of deliberate rumination, positive social responses, and meaning of life.

**Table 4:** Comparison between the indirect effects of deliberate rumination, positive social responses, and meaning of life.

Difference of indirect effects	Effect	*S.E.*	LLCI	ULCI
Deliberate rumination vs. positive social responses	−0.066	0.054	−0.1813	0.0327
Deliberate rumination vs. meaning of life	−0.048	0.042	−0.1322	0.0318
Positive social responses vs. meaning of life	0.018	0.055	−0.0890	0.1258

*LLCI, lower for confidence interval; ULCI, upper level for confidence interval*.

## Discussion

This study identified the relationships among self-disclosure, deliberate rumination, positive social responses, meaning of life, and the PTG of Korean adults, and examined a multiple mediating model of deliberate rumination, positive social responses, and meaning of life on self-disclosure and PTG. These attempts have produced academically and clinically significant results for PTG, and the implications are discussed below.

First, the frequency and severity of traumatic events is not significantly correlated with the PTG of Korean adults. This result reveals that experiencing repeated trauma or the existence of a high intensity of trauma does not automatically mean that psychological growth is achieved after experiencing trauma. This suggests that growth occurs when intra-psychological or behavioral processes occur after experiencing traumas, not as a result of the trauma itself. Therefore, this study informs internal and external psychological factors that cause PTG.

Self-disclosure, assumed to be a predictor or independent variable in this study, is positively correlated with PTG. It seems more logical that self-disclosure related to trauma could lead to growth rather than the assumption that growth after experiencing trauma increases self-disclosure. Some studies (e.g., Urry et al., 2011) *reported* that self-disclosure plays an important role in growth during childhood and adolescence, whereas this study found that self-disclosure might promote growth after the experience of trauma in adulthood. Pennebaker (2004) explained that self-disclosure helps to make sense of trauma experiences and to organize thoughts related to them. Therefore, we assumed that arranging and organizing trauma-related thoughts could be achieved through deliberate rumination, and this study demonstrated that self-disclosure was correlated with deliberate rumination.

This study also revealed that deliberate rumination was positively correlated with the PTG of Korean adults, and *d*eliberate rumination might have a cognitive effect, such as cognitive awareness (Laurenceau et al., 1998) and cognitive reconstruction (Huppert, 2009), that contributes to the healing or growth of people who have experienced trauma. In the parallel multiple mediating model assumed in this study, deliberate rumination mediates significantly between self-disclosure and PTG. The deliberate rumination caused by self-disclosure does result in growth after experiencing trauma for Korean adults. This finding not only reiterates the results of previous studies (Martin and Tesser, 1996; Treynor et al., 2003; Triplett et al., 2012; Ko and Rhee, 2018; Kim and Bea, 2019; Wozniak et al., 2020) that deliberate rumination can promote PTG, but also suggests that the deliberate rumination caused by self-disclosure can lead to PTG as well as recovery from PTSD. Therefore, this result makes the rationale, in encouraging patients with PTSD to engage in self-disclosure treatment in groups, more clinically persuasive. In addition, it suggests that deliberate rumination can also play an important role in PTG as well as PTSD prevention for the general population who have experienced traumatic events.

However, this study revealed that PTG is not only promoted in cognitive copings, because positive social responses have resulted in PTG. In fact, rumination and organizing thoughts related to traumas occurring after self-disclosure is an incidental effect, and the purpose of people telling others about themselves is likely to elicit empathy or receive emotional support for consolation. Chaudoir and Fisher (2010) support the assumption that if people receive social support, especially emotional support, they are likely to continue self-disclosure. Furthermore, self-disclosure is closely related to positive social responses in this study. These two variables share about 23% of the variation. In the multiple mediating model with deliberate rumination and meaning of life, positive social responses also significantly mediated the relationship between self-disclosure and PTG. This also means that positive social responses received from others after self-disclosure results in growth after experiencing trauma in Korean adults. There are also many emotional aspects to this effect, indicating that self-disclosure can result in in instilling emotional stability with positive social responses, which can lead to growth even after experiencing trauma. However, further studies are necessary to explore how positive social responses help people obtain PTG after experiencing traumatic events.

The variable most closely related to PTG in this study is the meaning of life. This means that the growth experienced after trauma is deeply related to realizing the meaning of life. Perhaps discovering the meaning of life is a condition of PTG. Triplett et al. (2012) examined the path through which people found the meaning of life after PTG through deliberate rumination; however, this study assumed that people would realize the meaning of life in the process of deliberately ruminating about the trauma experience. Furthermore, supporting Pennebaker (2004) assumption that self-disclosure makes people realize the meaning of life, self-disclosure and the meaning of life are significantly correlated in this study. In the multiple mediating model assumed in this study, meaning of life also mediates between self-disclosure and PTG significantly. Realizing the meaning of life during or after self-disclosure might lead Korean adults to PTG.

In the parallel multiple mediating model in this study, self-disclosure is not significantly accounted for the PTG of Korean adults. This means that the three psychological variables, deliberate rumination, the positive social responses, and meaning of life mediate between self-disclosure and PTG completely. The effect of self-disclosure on PTG is caused by these three variables. In addition, there are no significant differences in the magnitude of their effects in the model. These results have great implications in the clinical approach toward patients who have experienced trauma. It is necessary to focus on deliberate rumination, positive social responses, and discovering the meaning of life when encouraging patients with PTSD or people who have experienced trauma to share their experiences in a clinical setting. In fact, Yang and Ha (2019) found that firefighters’ PTG is likely to be promoted if their deliberate ruminations were enhanced after experiencing work-related traumatic events. Although, self-disclosing trauma may prevent post-traumatic stress symptoms (Stein et al., 2017), patient with PTSD can experience PTG if therapists help patients receive positive social responses from listeners and find meaning of life in the self-disclosing process. In addition, these three factors need to be considered when attempting to promote PTG even when applying other clinical methods, and not just for self-disclosure.

Because people are paying more attention to disasters and the personal trauma experiences they cause, this study investigated some psychological variables that could promote growth even after traumatizing experience, and these yielded academically and clinically useful information. However, there are some deficiencies and limitations in interpreting the results of this study. First, the sample used in this study is not representative of the Korean adult population because the data were collected with convenience sampling. Therefore, further studies are necessary to re-verify the relationship between the variables revealed in this study. Second, the process of collecting data online resulted in high proportion of women among the participants. This could probably be justified because the data collection process was not artificially controlled. In fact, the prevalence of anxiety disorders and PTSD is high in women (American Psychiatric Association, 2013). In addition, the Korea National Insurance Services (2020) reported that the number of female patients with PTSD in their 20s has increased 2.1 times over the past 5 years, and the overall number of female patients treated for PTSD was about 1.5 times that of male patients. Finally, the cause-and-effect relationship was discussed based on the results of previous studies and logic; however, the cause-and-effect relationship cannot be completely concluded based on the results obtained from a correlational study, not an experimental study.

## Conclusion

This study investigated the relationship between self-disclosure and the PTG of Korean adults, and examined a parallel multiple mediating model of deliberate rumination, positive social responses, and meaning of life on self-disclosure and PTG. Results of the correlational analysis revealed that deliberate rumination, positive social responses, and meaning of life, as well as self-disclosure are positively correlated with the PTG of Korean adults. Furthermore, self-disclosure is positively correlated with deliberate rumination, positive social responses, and meaning of life. Positive social responses are closely correlated with self-disclosure, whereas the meaning of life is closely correlated with the PTG of Korean adults.

In the multiple mediating model, self-disclosure positively affected deliberate rumination, and deliberate rumination could promote people’s PTG, whereas self-disclosure also affects positive social responses, which could improve PTG. Additionally, self-disclosure influences meaning of life positive, and meaning of life could increase PTG. In this model, deliberate rumination, positive social responses, and meaning of life mediate between self-disclosure and PTG completely, whereas there are no significant differences in the magnitude of the mediating effect of these three variables. Finally, it is expected that this study’s findings will provide valuable information for future research and for mental health professionals attempting to not only prevent PTSD of persons who experienced traumatic events but also to promote the PTG of their patients with PTSD.

## Data Availability Statement

The raw data supporting the conclusions of this article will be made available by the authors, without undue reservation.

## Ethics Statement

The studies involving human participants were reviewed and approved by Institutional Review Board of Sahmyook University (code: 2-1040781-A-N-01). The patients/participants provided their written informed consent to participate in this study.

## Author Contributions

J-HR designed the study, collected data, and conducted the literature review. K-HS led analyzed and interpreted the data and wrote the final manuscript. All authors contributed to the article and approved the submitted version.

## Conflict of Interest

The authors declare that the research was conducted in the absence of any commercial or financial relationships that could be construed as a potential conflict of interest.

## Publisher’s Note

All claims expressed in this article are solely those of the authors and do not necessarily represent those of their affiliated organizations, or those of the publisher, the editors and the reviewers. Any product that may be evaluated in this article, or claim that may be made by its manufacturer, is not guaranteed or endorsed by the publisher.

## Abbreviations

IRB: Institutional review board
MLQ: Meaning in life questionnaire
SRQ: Social reactions questionnaire
VIF: Variance inflation factors
PTG: Post-traumatic growth
PTSD: Post-traumatic stress disorder
SPSS: Statistical package for social sciences
